# Exploiting Real-Time Genomic Surveillance Data To Assess 4CMenB Meningococcal Vaccine Performance in Scotland, 2015 to 2022

**DOI:** 10.1128/mbio.00499-23

**Published:** 2023-04-10

**Authors:** C. M. C. Rodrigues, L. MacDonald, R. Ure, A. Smith, J. C. Cameron, M. C. J. Maiden

**Affiliations:** a Department of Biology, University of Oxford, Oxford, United Kingdom; b Department of Infection Biology, London School of Hygiene and Tropical Medicine, London, United Kingdom; c Department of Paediatrics, Imperial College Healthcare NHS Trust, London, United Kingdom; d Public Health Scotland, Glasgow/Edinburgh, United Kingdom; e Bacterial Respiratory Infection Service, Scottish Microbiology Reference Laboratory, Glasgow Royal Infirmary, Glasgow, United Kingdom; f College of Medical, Veterinary & Life Sciences, Glasgow Dental Hospital & School, University of Glasgow, Glasgow, United Kingdom; LMU Munich

**Keywords:** vaccination, Bexsero, 4CMenB, breakthrough cases, genomics, WGS, MenDeVAR, BAST, meningococcal infections

## Abstract

The United Kingdom implemented the first national infant immunization schedule for the meningococcal vaccine 4CMenB (Bexsero) in September 2015, targeting serogroup B invasive meningococcal disease (IMD). Bexsero contains four variable subcapsular proteins, and postimplementation IMD surveillance was necessary, as nonhomologous protein variants can evade Bexsero-elicited protection. We investigated postimplementation IMD cases reported in Scotland from 1 September 2015 to 30 June 2022. Patient demographics and vaccination status were combined with genotypic data from the causative meningococci, which were used to assess vaccine coverage with the meningococcal deduced vaccine antigen reactivity (MenDeVAR) index. Eighty-two serogroup B IMD cases occurred in children >5 years of age, 48 (58.5%) of which were in unvaccinated children and 34 (41%) of which were in children who had received ≥1 Bexsero dose. Fifteen of the 34 vaccinated children had received one dose, 17 had received two doses, and two had received three doses. For 39 cases, meningococcal sequence data were available, enabling MenDeVAR index deductions of vaccine-preventable (M-VP) and non-vaccine-preventable (M-NVP) meningococci. Notably, none of the 19 of the children immunized ≥2 times had IMD caused by M-VP meningococci, with 2 cases of NVP meningococci, and no deduction possible for 17. Among the 15 children partially vaccinated according to schedule (1 dose), 7 were infected by M-VP meningococci and 2 with M-NVP meningococci, with 6 for which deductions were not possible. Of the unvaccinated children with IMD, 40/48 were ineligible for vaccination and 20/48 had IMD caused by M-VP meningococci, with deductions not being possible for 14 meningococci.

## INTRODUCTION

Neisseria meningitidis, the meningococcus, occurs widely as a member of a healthy human microbiota. Meningococci are highly diverse, however, and some variants have a propensity to invade host tissues (1), causing septicemia and/or meningitis. Invasive meningococcal disease (IMD) has a 5 to 6% mortality (2), with a third of survivors developing sequelae (3). In many settings, IMD incidence is highest in infancy (children <1 year old), followed by those aged 1 to 5 years (4). Current vaccines include adjuvanted capsular polysaccharides (5) covering up to five of the six disease-associated capsule variants (serogroups A, C, W, X, and Y). For serogroup B, a major cause of IMD in many countries, capsular vaccines have not been developed as a consequence of safety and efficacy concerns (6), and vaccine development has focused on protein antigens, e.g., the 4CMenB vaccine (Bexsero [GlaxoSmithKline, Siena, Italy], licensed in 2013) (7) and the rLP4086 vaccine (Trumenba [Pfizer, New York, USA], licensed in 2015) (8).

The Bexsero vaccine combines the outer membrane vesicle (OMV) vaccine MeNZB, developed for an IMD outbreak in New Zealand (7), with three recombinant proteins: factor H binding protein (fHbp), neisserial heparin binding antigen (NHBA), and *Neisseria* adhesin A (NadA). The bivalent Trumenba vaccine contains two fHbp variants (8). Most meningococcal major outer membrane proteins are immunogenically highly diverse (9), which is important when these proteins are used as vaccine components, as vaccine-elicited immune responses, especially antibodies, may not recognize different variants. At the population level, the similarity of the variants in the vaccine formulation to those among IMD-associated meningococci in different geographical regions is important for vaccine policy decisions. For example, Bexsero was estimated to prevent 66% of serogroup B IMD cases in England and Wales in the epidemiological year 2014/15 (the epidemiological year is from 1 July to 30 June) (10), which contributed to national implementation on 1 September 2015. A two-dose priming schedule at 2 and 4 months of age with a booster at 12 months was used, with children born between 1 September 2015 and 30 June 2015 being eligible for single catch-up immunizations. Bexsero vaccine uptake in Scotland was high: annual rates were >95.4% for the primary course and 93.5% for the booster dose (11).

Postimplementation surveillance of Bexsero vaccine effectiveness includes assessing the reactivity of vaccine-derived antibody responses with representative contemporary variant meningococci, either by phenotypic assays or by deduction from genotype. Assays include the meningococcal antigen typing system (MATS) (12) for Bexsero and the meningococcal antigen surface expression (MEASURE) (13) assay for Trumenba. Both are time and resource intensive and must be performed at specialist laboratories, which hinders real-time surveillance. Molecular approaches are increasingly available for routine microbiology, enabling population-scale surveillance by means of real-time whole-genome sequencing (WGS). The Bexsero antigen sequence typing (BAST) scheme (14) catalogues variants of Bexsero antigens for such surveillance. BAST analysis suggested vaccine coverage of 60 to 66% of all meningococci and 58% of genogroup B meningococci from 2010 to 2016 (15). The meningococcal deduced vaccine antigen reactivity (MenDeVAR) index combines genotypic data with BAST and phenotypic data, i.e., published MATS, MEASURE, and serum bactericidal activity (SBA) assays. For any given meningococcal isolate, this indicates likely vaccine coverage for Bexsero and Trumenba (16), providing actionable public health information for vaccine use in outbreak/cluster settings and surveillance. The Bacterial Respiratory Infection Service, Scottish Microbiology Reference Laboratory, Glasgow (SMiRL-G), introduced real-time WGS in 2018, and these data were used to assess serogroup B meningococci in children in Scotland <5 years old, including potential vaccine breakthrough cases, to help understand the IMD epidemiology in the post-Bexsero vaccine implementation period.

## RESULTS

**FIG 1:**
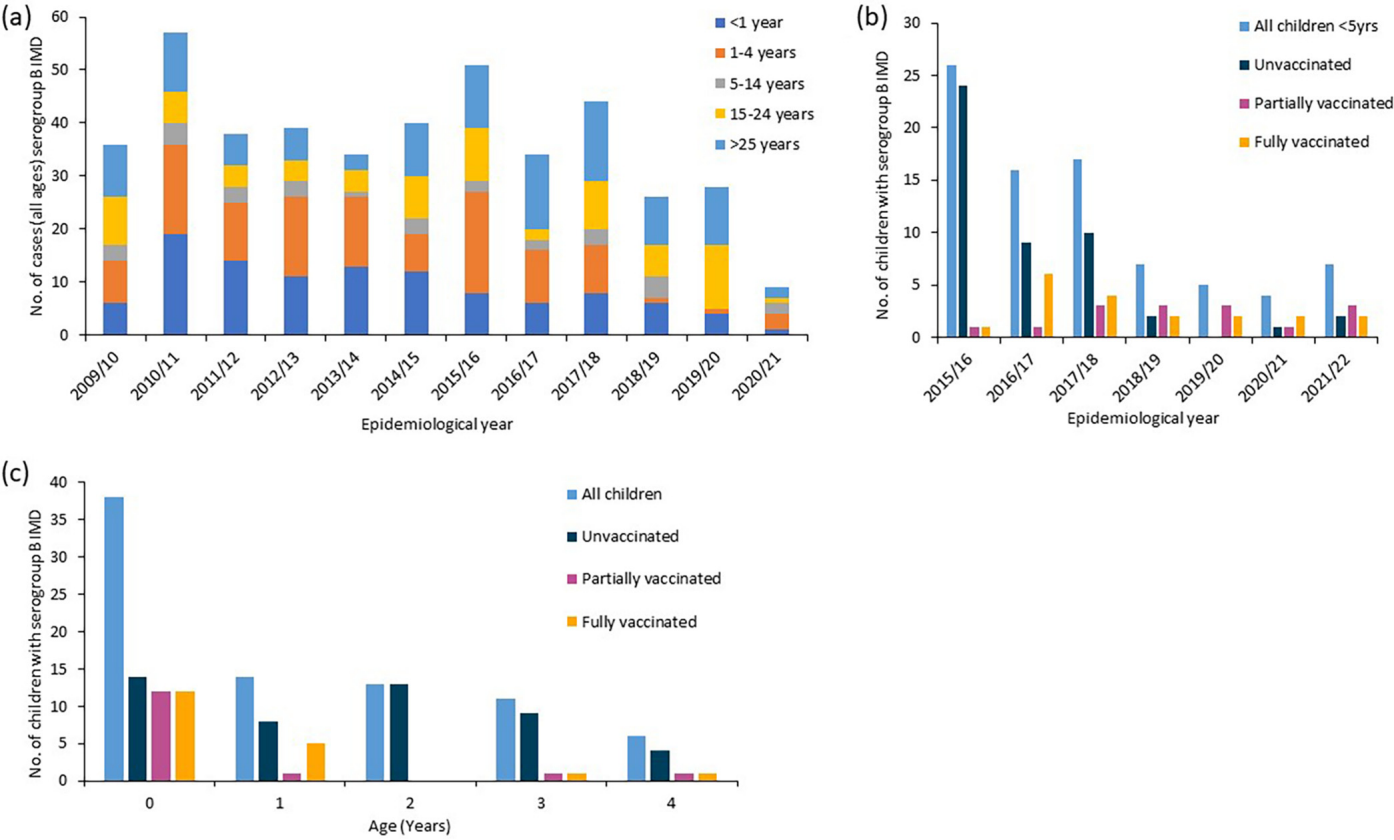
(a) IMD serogroup B cases in Scotland from 2008/09 to 2021/22 by age group. (b) Distribution of IMD cases in <5-year-old children from 2015/16 to 2021/22. (c) Distribution of IMD cases in <5-year-old children from 2015 to 2022. The epidemiological year is from 1 July to 30 June. Children were deemed fully or partially vaccinated according to the UK immunization schedule.

A total of 126 IMD cases were reported after vaccine implementation, i.e., from 1 September 2012 to 30 June 2022. Of these, 82 (65.1%) were caused by serogroup B organisms, with microbiological data available (Fig. 1a and b), 39 (47.6%) of which were diagnosed by positive blood/cerebrospinal fluid (CSF) culture and 43 (52.4%) by meningococcal PCR (blood/CSF). Of the 43 PCR-positive samples, further typing was possible for 27 (62.8%) for fHbp and 29 (67.4%) for PorA, and for 12 (27.9%), the MenDeVAR index determined an exact or cross-reactive match. Overall, for 14.6% (*n* = 12) of all IMD cases, no typing data were available.

Of the 82 children with serogroup B IMD, 48 (58.5%) had not received any doses of Bexsero (referred to here as unvaccinated), of whom 36 (75.0%) were born before implementation and too old for the catch-up program, 4 (8.3%) were born after July 2015 but developed IMD before the first 8-week dose, and 8 (16.7%) were eligible but not vaccinated. Of the IMD patients, 34/82 (41.5%) had received ≥1 dose of Bexsero, 15 (18.3%) of whom were partially vaccinated according to schedule (1 dose, 11 [13.4%]; 2 doses, 4 [4.9%]). Nineteen (23.2%) were fully vaccinated according to schedule (2 doses, 17 [20.7%]; 3 doses, 2 [2.4%]). For the 28 patients born after 1 September 2015 who had received ≥1 dose of Bexsero, median age at first dose was 9 weeks (range, 8 to 12 weeks) and median age at second dose was 18 weeks (range, 17 to 20 weeks); only one child received a booster dose at 13 months.

The 82 IMD cases occurred across all seven epidemiological years, 2015/16 to 2021/22 (Fig. 1a and c), but incidence decreased over time, with most occurring in the first three epidemiological years (Table 1), when cases in unvaccinated children predominated. The cohort of children born from May 2015 were fully vaccinated at high rates. Clinical presentation was known for 77 cases (94%): septicemia (35 [42.7%]), meningitis (24 [29.3%]), both meningitis and septicemia (17 [20.7%]), and septic arthritis (1 [1.2%]) (Table 1).

**TABLE 1:** Characteristics of children who developed IMD, grouped by vaccination status^*a*^

Characteristic	No. (%) of children			
	Unvaccinated	Partially vaccinated	Fully vaccinated	Total
Epidemiological yr				
2015/16	24 (50.0)	1 (6.7)	1 (5.3)	26 (31.7)
2016/17	9 (18.8)	1 (6.7)	6 (31.6)	16 (19.5)
2017/18	10 (20.8)	3 (20.0)	4 (21.1)	17 (20.7)
2018/19	2 (4.2)	3 (20.0)	2 (10.5)	7 (8.5)
2019/20	0 (0.0)	3 (20.0)	2 (10.5)	5 (6.1)
2020/21	1 (2.1)	1 (6.7)	2 (10.5)	4 (4.9)
2021/22	2 (4.2)	3 (20.0)	2 (10.5)	7 (8.5)
Age (yrs)				
0	14 (29.2)	12 (80.0)	12 (63.2)	38 (46.3)
1	8 (16.7)	1 (6.7)	5 (26.3)	14 (17.1)
2	13 (27.1)	0 (0.0)	0 (0.0)	13 (15.9)
3	9 (18.8)	1 (6.7)	1 (5.3)	11 (13.4)
4	4 (8.3)	1 (6.7)	1 (5.3)	6 (7.3)
Clinical presentation				
Septicemia	21 (43.8)	5 (33.3)	9 (47.4)	35 (42.7)
Meningitis	16 (33.3)	3 (20.0)	5 (26.3)	24 (29.3)
Meningitis/septicemia	9 (18.8)	6 (40.0)	2 (10.5)	17 (20.7)
Septic arthritis	0 (0.0)	0 (0.0)	1 (5.3)	1 (1.2)
Unknown	2 (4.2)	1 (6.7)	2 (10.5)	5 (6.1)
Microbiology				
Culture confirmed	17 (35.4)	11 (73.3)	11 (57.9)	39 (47.6)
PCR confirmed	31 (64.6)	4 (26.7)	8 (42.1)	43 (52.4)
Total	48	15	19	82

a Children were defined as being fully vaccinated according to schedule if they had been fully vaccinated with Bexsero for their age and ≥14 days had passed between the last Bexsero dose and the date of IMD onset. Children were defined as partially vaccinated according to schedule if they had received vaccine doses appropriate for age but developed IMD <14 days after the last Bexsero dose or they were overdue for subsequent vaccine doses. Children were defined as unvaccinated if they had never received a dose of Bexsero or if there were <14 days between the first dose of Bexsero and the date of IMD onset.

**FIG 2:**
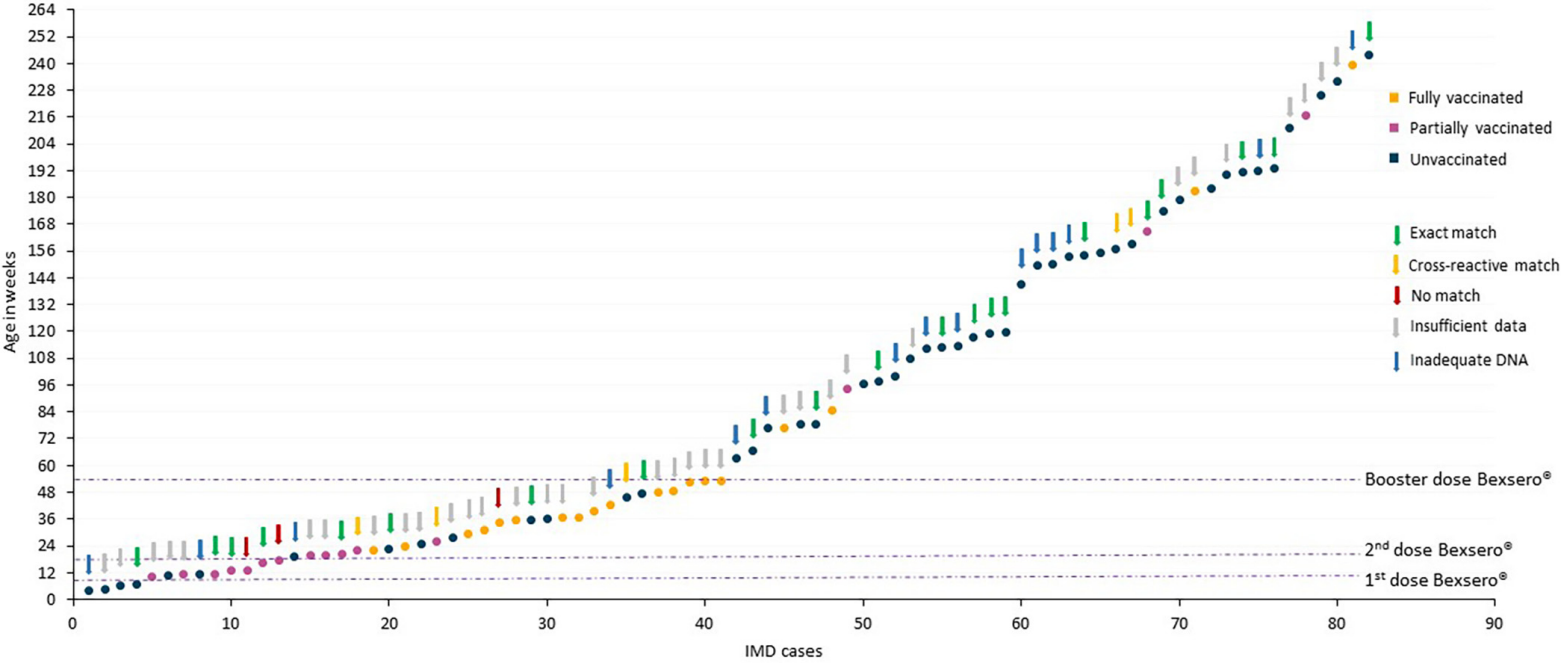
Distribution of all IMD cases in children <5 years old in Scotland from September 2015 to June 2022. Cases are shown with dots by increasing age at onset of IMD, measured in weeks. The dots are colored by vaccination status (yellow, fully vaccinated; purple, partially vaccinated; blue, unvaccinated). For each case, the MenDeVAR index for the invasive meningococcal isolate is shown by the arrow above the dot, colored green for an exact match to vaccine variants, amber for a cross-reactive match to vaccine variants, red for no match to vaccine variants, gray where there were insufficient data to interpret reactivity, or blue where there was inadequate DNA to determine an antigenic profile for PCR-confirmed cases. The timing of the 2 + 1 Bexsero dosing schedule is shown with the yellow dotted line, with the first dose at 8 weeks, the second dose at 16 weeks, and the booster dose at 12 months. Children were deemed fully or partially vaccinated according to the UK immunization schedule.

There were 38/82 (46.3%) IMD cases among infants, the highest-risk group (Table 1). These were distributed among infants who were unvaccinated (14/38 [36.8%]), partially vaccinated according to schedule (1 dose) (12/38 [31.6%]), and fully vaccinated according to schedule (2 doses) (12/38 [31.6%]). Of the 14 cases in unvaccinated infants, four occurred between 4 and 7 weeks of age, prior to the first dose (8 weeks) (Fig. 2). The remaining 10 infants were between 11 and 46 weeks old at IMD onset. Of the 12 infants who received one Bexsero dose, six developed IMD before the second dose at 16 weeks and the remaining six were overdue for their second priming dose by 4 to 10 weeks when they developed IMD. Among the 12 infants who were fully vaccinated according to schedule (2 doses), IMD developed between 22 and 48 weeks of age.

Cases were less frequent in children >1 year old who had received any vaccine doses (10/44 [22.7%]) than in those with no vaccine (34/44 [77.3%]) (χ^2^ = 13.73; *P* = 0.0002) (Table 1; Fig. 2). Among the 10 children >1 year old who had received ≥1 vaccine dose and developed IMD, 8 had not received their 12-month booster dose, with 3 being only days past their first birthday, and 2 developed IMD at 3 to 4 years old, despite completing a 2 + 1 Bexsero schedule. The time between the last dose of vaccine and IMD onset was highly variable: the time after the first dose was 16 to 110 days, that after the second dose was 7 to 1,029 days, and that after the third dose was 875 to 1,235 days.

**FIG 3:**
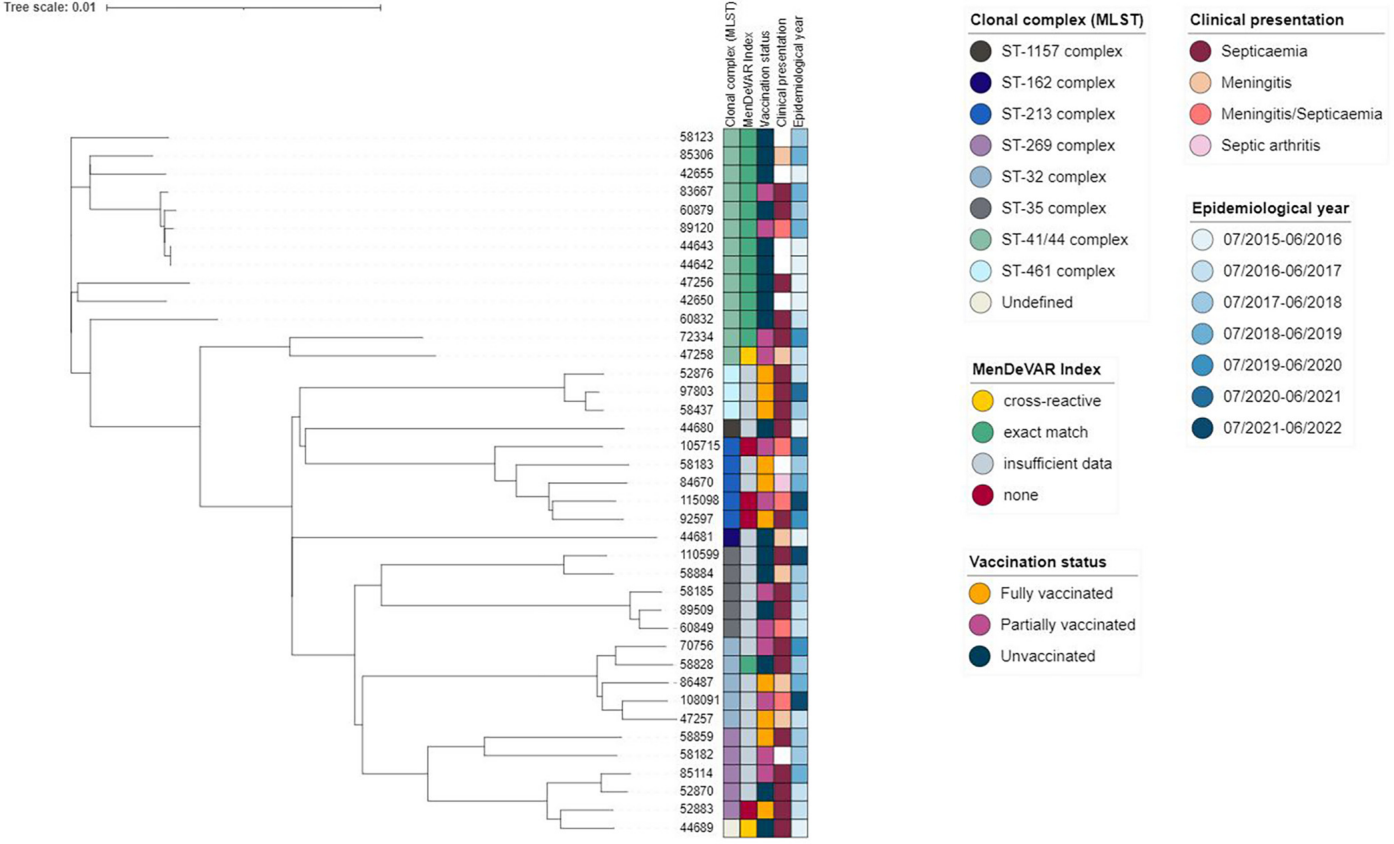
Phylogenetic relationships of IMD-causing meningococci in children <5 years old isolated from culture-confirmed disease. Unrooted phylogeny generated using allele-based core genome MLST (cgMLST), with the PubMLST ID at the end of the branches. Relevant genotypic and phenotypic data are shown in colored bars. Children were deemed fully or partially vaccinated according to the UK immunization schedule.

WGS data were available for 39/82 cases and provided the clonal complex (CC) distribution and the predicted MenDeVAR index of the invasive meningococci, which could be compared to vaccination status for each of the IMD cases (Fig. 3). Most cases considered vaccine preventable were caused by CC41/44 meningococci (13/15 [86.7%]), predominantly in unvaccinated children (9/13 [69.2%]). The antigens responsible for the predicted vaccine coverage in these cases were NHBA peptide 2 (*n* = 8, exact match), peptide 10 (*n* = 2, cross-reactive match), and PorA variable region 2 (VR2) 4 (*n* = 6, exact match), with 3 isolates possessing 2 of these antigens.

**FIG 4:**
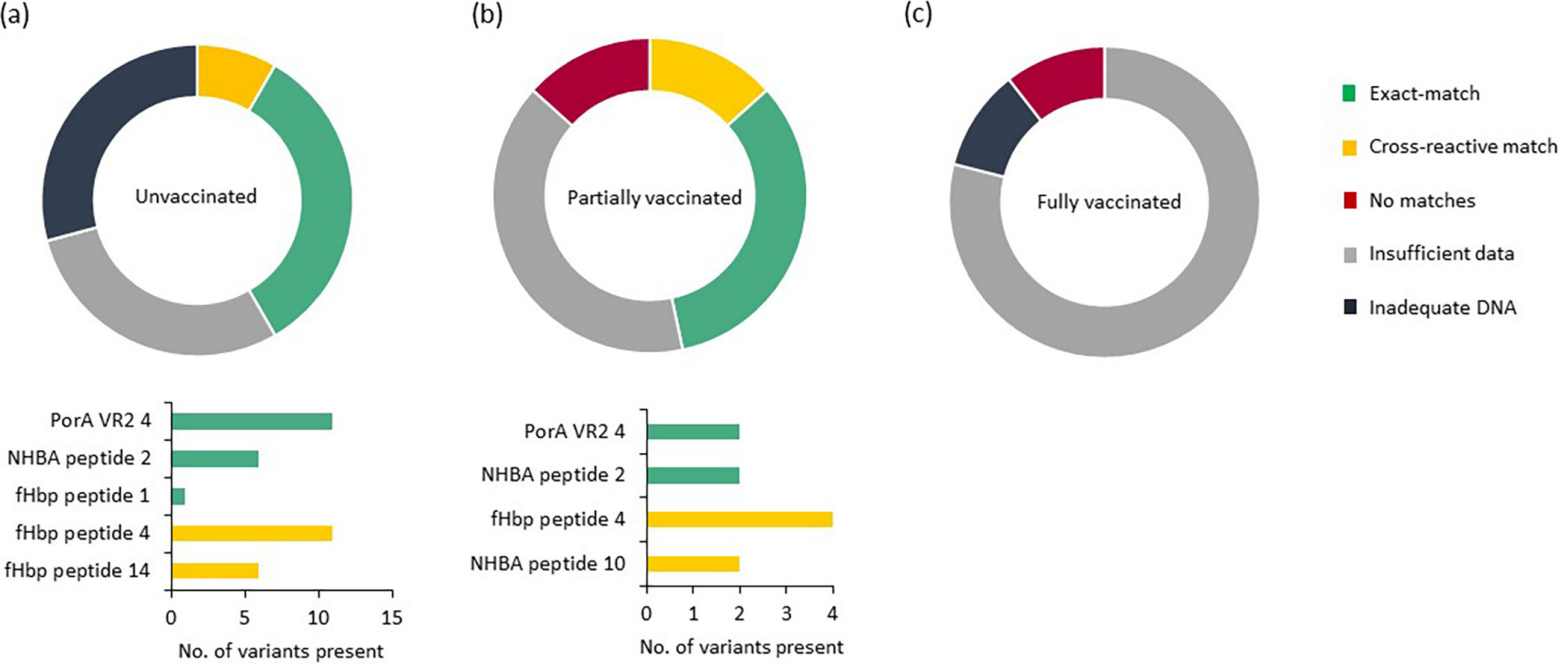
MenDeVAR index output for IMD cases for each group of children considering vaccination status. Segments of the pie chart are colored according to matches to vaccine variants. Bar charts at the bottom demonstrate the frequency of peptide variants present in the isolates that were deemed to be vaccine preventable. The number of variants is greater than the number of isolates, as some isolates possess multiple vaccine-reactive antigens; 4/7 meningococci isolated from unvaccinated individuals and 14/20 meningococci isolated from partially vaccinated individuals had ≥1 potentially cross-reactive/exact antigen. Children were deemed fully or partially vaccinated according to the UK immunization schedule.

Of the 48 IMD cases among unvaccinated children (Fig. 2), 17 (40%) were culture confirmed and 31 (74%) were PCR confirmed, with 21 (42%) of these having typing information; however, multilocus sequence type (MLST) was not determined for any PCR-confirmed isolates. Clonal complex designations of the 17 meningococci with WGS data were as follows: CC41/44, 9 isolates; CC35, 3; CC162, 1; CC1157, 1; CC269,1; CC32, 1; no CC defined, 1 (sequence type 1159 [ST-1159]) (Fig. 3). The fHbp peptide was detected in 34 isolates and designated by (i) Novartis subfamily (subfamily 1, 26 isolates; subfamily 2, 6; and subfamily 3, 2) and (ii) Pfizer (subfamily A, 8 isolates, and subfamily B, 26). The most frequent fHbp peptides were 4 (11 isolates), 14 (6 isolates), and 13 (4 isolates). Vaccine variant peptide 1 was present in one isolate. PorA VR1 and PorA VR2 data were available for 36 (75%) meningococci, and 12 specimens were unsuitable for further analysis. Eleven meningococci possessed the PorA P1.4 VR2 variant present in the vaccine. NHBA was present in all cultured meningococci, and six of these possessed vaccine variant peptide 2. NadA was present in 1 cultured meningococcus with peptide 1, which is not reactive with vaccine-induced immune responses. The MenDeVAR index predicted 20 cases as vaccine preventable, with 14/20 possessing ≥1 antigen potentially cross-reactive with vaccine-induced immune responses (Fig. 4a). Fourteen isolates had insufficient data; i.e., they possessed fHbp, NHBA, and NadA variants that had not been experimentally tested. Such testing would enable future prediction of their antigenic phenotype from the genotype.

Fifteen IMD cases occurred in children who were partially vaccinated according to schedule, of which 11 were culture confirmed and four PCR confirmed with vaccine antigen typing. For 12 cases, MLST was determined: CC41/44, 6 isolates; CC35, 1; C32, 2; CC213, 2; and CC269, 1 (Fig. 3). The fHbp peptide was present in all isolates and categorized in Novartis subfamilies (subfamily 1, 7 isolates; subfamily 2, 4; and subfamily 3, 4) and Pfizer subfamily (subfamily A, 8 isolates, and subfamily B, 7). The most frequent fHbp peptide was peptide 4, found in 4 meningococci, and vaccine variant peptide 1 was not present. PorA VR1 and PorA VR2 were present in all organisms, with two (18.2%) possessing vaccine variant P1.4. NHBA was present in all 11 meningococci with WGS data, with vaccine variant NHBA peptide 2 in 3/11 (27.2%) organisms. NadA was present in 1/11 (9.1%). The MenDeVAR index predicted no cross-reactivity for two isolates, both children received 1 dose (Fig. 4b). Seven children with IMD were infected by meningococci considered to be vaccine-preventable, five meningococci with exact matches (4 received 1 dose, and 1 received 2 doses) and two with cross-reactive (both received 2 doses) antigen variants. Six specimens had insufficient data to facilitate prediction of phenotype.

Nineteen IMD cases occurred across all seven epidemiological years in children who were fully vaccinated according to schedule: 12 in infants, five in toddlers 1 to 2 years old, and two in 3- to 5-year-olds (Table 1). Of these, 11 were culture confirmed and eight were PCR confirmed (Table 2). For 13 cases, MLST profiles of the invasive meningococci were determined (CC213, 4 isolates; CC461, 3; CC32, 2; CC269, 2; CC35, 1; and CC41/44, 1) (Fig. 3). The fHbp peptide was determined for 17 meningococci and categorized into Novartis subfamilies (subfamily 1, 4 isolates; subfamily 2, 4; and subfamily 3, 9) and Pfizer subfamily (subfamily A, 4 isolates, and subfamily B, 13). The commonest fHbp peptides were 13 (3 isolates) and 47 (3 isolates). PorA VR1 and PorA VR2 were identified in 15/17 specimens. NHBA was present in 11 isolates with WGS data and NadA in 3 of these (Table 2). The MenDeVAR index predicted no cross-reactivity for two meningococci; both children received 2 doses (Fig. 4c). No meningococci had an exact or cross-reactive match with Bexsero antigens, and 15 isolates had insufficient data for phenotype prediction. Among these were variants for which insufficient MATS data were available, including fHbp peptides 13 and 321 and NHBA peptides 3, 21, 115, and 118. Children who were fully vaccinated according to schedule were less likely to develop IMD with vaccine preventable strains (0/19 [0.0%]), compared to unvaccinated children (20/48 [22.7%]) (χ^2^ = 14.08; *P* = 0.0002).

**TABLE 2:** Detailed genomic typing of the 19 Bexsero breakthrough cases in Scotland from 2015 to 2022, occurring in children <5 years old who were fully vaccinated

Epidemiological yr	Clinical presentation	PubMLST ID^*a*^	Age (yrs)	No. of doses	Days from last dose to IMD onset	ST (MLST)	CC	Peptide			PorA VR		MenDeVAR reactivity		BAST
								fHbp	NHBA	NadA	1	2	Bexsero	Trumenba	
2015/16	Septicemia	PCR	0	2	133	ND	ND	95	ND	ND	21-2	28	Insufficient data	Insufficient data	ND
2016/17	Meningitis	47257	0	2	89	32	ST-32 complex	29	3	100	7-11	16-44	Insufficient data	Insufficient data	2138
2016/17	Septicemia	2876	0	2	211	1946	ST-461 complex	47	118	0	19-2	13-1	Insufficient data	Cross-reactive	230
2016/17	Septicemia	52883	0	2	143	275	ST-269 complex	19	31	0	22	9	None	Cross-reactive	2378
2016/17	Meningitis/Septicemia	60849	1	2	313	2380	ST-35 complex	95	29	0	21-2	28	Insufficient data	Insufficient data	290
2016/17	Meningitis	PCR	0	2	181	ND	ND	Inadequate DNA	Inadequate DNA	Inadequate DNA	Inadequate DNA	Inadequate DNA	ND	ND	ND
2016/17	Septicemia	PCR	1	2	182	ND	ND	1143	ND	ND	22	14	Insufficient data	Insufficient data	ND
2017/18	Septicemia	58859	0	2	20	1416	ST-269 complex	13	21	0	5	2	Insufficient data	Cross-reactive	2946
2017/18	Unknown	58183	0	2	159	6607	ST-213 complex	13	117	21	22	14	Insufficient data	Cross-reactive	1812
2017/18	Septicemia	58437	0	2	120	1946	ST-461 complex	47	118	0	19-2	13-1	Insufficient data	Cross-reactive	230
2017/18	Septicemia	PCR	0	2	224	ND	ND	13	D	+	22	9	Insufficient data	Cross-reactive	
2018/19	Joint Infection	84670	0	2	31	213	ST-213 complex	499	115	122	18-1	25	Insufficient data	Insufficient data	3079
2018/19	Meningitis	86487	3	3	875	32	ST-32 complex	174	3	0	7	16	Insufficient data	Insufficient data	3180
2019/20	Septicemia	92597	0	2	106	9193	ST-213 complex	45	18	0	22	14	None	Exact match	224
2019/20	Unknown	PCR	4	3	1,235	ND	ND	Inadequate DNA	Inadequate DNA	ND	Inadequate DNA	Inadequate DNA	Inadequate DNA	Inadequate DNA	ND
2020/21	Meningitis/Septicemia	PCR	1	2	230	ND	ND	321	ND	ND	5-2	10-1	Insufficient data	Insufficient data	ND
2020/21	Septicemia	97803	1	2	230	15683	ST-461 complex	47	118	0	19-2	13-1	Insufficient data	Cross-reactive	230
2021/22	Meningitis	PCR	0	2	89	9193	ST-213 complex	45	ND	ND	22	14	Insufficient data	Exact match	ND
2021/22	Meningitis	PCR	1	2	252	1414	ST-41/44 complex	19	ND	ND	18-1	1	Insufficient data	Cross-reactive	ND

a Where the isolate was culture confirmed, the meningococci were subjected to whole-genome sequencing, and so a genome record is present on pubmlst.org and can be found using the PubMLST ID. For each isolate, culture confirmed or PCR confirmed, the typing data for ST, CC, fHbp, NHBA, NadA, and PorA VR1 and VR2 are listed where available. NHBA and NadA testing was not routinely performed as part of NHS workflow at the time of the study. ND, not determined; PorA VR, PorA variable region.

## DISCUSSION

These data demonstrate how genomic surveillance of a variable bacterial pathogen, which can be achieved in real time or nearly real time, can support vaccination implementation. In 2015, the United Kingdom was the first country to introduce the protein-based meningococcal vaccine Bexsero into a national infant immunization program, although its efficacy against all meningococcal variants causing IMD was uncertain at that time. Vaccine uptake was high in Scotland, at 94.5 to 96.9% for the 2-dose priming course and 91.4 to 95.0% for the booster dose. Routine real-time genomic surveillance of IMD in Scotland (2015 to 2022) enabled the MenDeVAR index to be used. This demonstrated the effectiveness of Bexsero in fully vaccinated children, who experienced no IMD cases caused by vaccine-preventable meningococci, i.e., those with antigen variants that either were identical to those in the vaccine or had been shown in *in vitro* assays to be reactive to vaccinee sera (cross-reactive variants) (12, 16). However, some individuals who received a single dose of Bexsero did develop vaccine-preventable IMD.

There were 19 IMD cases in children who were fully vaccinated according to schedule, two of whom developed IMD caused by meningococci regarded as not preventable and therefore not anticipated to be covered by the Bexsero immunization program. The remaining 17 cases in the group who were fully vaccinated according to schedule (89%) had antigenic variants not tested by the MATS assay at the time of writing, precluding the determination of their vaccine-preventable status using the MenDeVAR index. A lasting protective effect of the booster dose was observed in children who were fully vaccinated according to schedule, with low numbers of cases in 3- and 4-year-old children who had received three doses and developed IMD (*n* = 2), supporting previous efficacy studies (17). These data therefore support the 2 + 1 schedule for priming advised by the UK Joint Committee of Vaccination and Immunization, rather than the 3 + 1 schedule from clinical trials (18, 19). Further postimplementation immunogenicity data may explain these observations in vaccinees, with >97% of infants who received a 2 + 1 schedule achieving a >4-fold rise in human SBA titers after both primary and booster doses (20). Bexsero protects children through the period of highest risk, from infancy to 5 years old, against certain meningococcal variants, and this should be communicated to parents and health care professionals; however, the occurrence of breakthrough cases with nonpreventable meningococci indicates that those caring for vaccinated children should remain alert for the signs and symptoms of suspected IMD and manage them with appropriate urgency.

IMD occurred most frequently in unvaccinated and partially vaccinated children (76.8%), with 17.1% of children who developed IMD not receiving all or any of the doses for which they were eligible. For 37.5% (3/8) of eligible but unvaccinated children and 46.7% (7/15) of children partially vaccinated according to schedule, their IMD was caused by a vaccine-preventable meningococcus. Although these numbers of individuals are small, the impact of IMD on children and families is often profound and long-lasting (3). These data suggest that vaccination is highly protective against diverse meningococcal variants, perhaps as many as 88% of meningococci (10, 21, 22); therefore, if universally applied, the existing schedule might have prevented up to half of IMD cases in eligible children <5 years old. Most partially vaccinated children were infants, of whom five had not received their second dose (due at 16 weeks) by 18 to 26 weeks of age, and three of these developed IMD with meningococci characterized as vaccine preventable. This supports the need for at least two priming doses, as one is likely insufficient to generate protective immunity. For the five children >1 year old who had received their priming course but not their boosters by 20 to 48 months, it was not possible to determine whether their meningococcal variants could have been prevented by adequate vaccination, as their antigens had not been tested in the MATS assay. Childhood vaccinations can be delayed for many reasons, including concurrent febrile illness, vaccination appointment delays, concern over side effects, and vaccine hesitancy or refusal. This was compounded by the severe acute respiratory syndrome coronavirus 2 (SARS-CoV-2) pandemic, resulting in reduced vaccine uptake in England during the first UK lockdown compared to prepandemic levels (23), although Scotland had an increase in timely vaccine uptake (24, 25). Refocusing efforts on positive and sustained messaging to carers and encouraging proactive identification and contact of unvaccinated children would help to prevent such cases.

Known hyperinvasive meningococcal lineages were responsible for IMD among Scottish children, reflecting the circulating meningococcal population causing disease in unvaccinated children and adults (see Fig. S1 in the supplemental material). There was no evidence of CC41/44 causing IMD in fully vaccinated children, consistent with the fact that the OMV component in Bexsero originates from the MeNZB vaccine, which is based on a CC41/44 meningococcus and therefore contains the characteristic PorA P1.7-2,4 variant as a dominant antigen. In post-MeNZB introduction surveillance in New Zealand in 2008, 34 vaccine breakthrough cases occurred in persons <19 years old caused by the epidemic PorA variant P1.7-2,4 meningococcus, with half occurring in children <5 years old (26). We did not observe any breakthrough cases with the PorA variant P1.7-2,4 or any CC41/44 meningococci. Of the CC41/44 isolates that did occur in unvaccinated and partially vaccinated Scottish children, 81% possessed at least two reactive fHbp, NHBA, or PorA VR2 antigens, suggesting that in Bexsero vaccinees, multiple antigens may result in enhanced protection rather than relying on the PorA P1.7-2,4 antigen alone for CC41/44.

10.1128/mbio.00499-23.1 FIG S1 GrapeTree analysis using cgMLST, showing the population structure of the 129 meningococcal genogroup B disease isolates from culture-confirmed cases of all ages in Scotland from 2014/15 to 2021/22. This represented 51.8% of all IMD over that period. Isolates from children <5 years old included in the analysis are circled in yellow, indicating the wider diversity of IMD. Download FIG S1, TIF file, 0.1 MB. Copyright © 2023 Rodrigues et al. 2023 Rodrigues et al. https://creativecommons.org/licenses/by/4.0/ This content is distributed under the terms of the Creative Commons Attribution 4.0 International license.

Postimplementation vaccine surveillance is required to monitor ongoing trends in disease-causing meningococci, including secular changes in lineages and vaccine antigen prevalence. Until 2016, this was performed through phenotypic assays, but with the development and application of genomic typing, particularly WGS, in public health laboratories, genomic surveillance has become achievable. Genomic data allow assessment of genetic population structure and detailed characterization of certain bacterial features, including capsular type and protein vaccine antigens. Furthermore, deduction of peptide variants from genomic data enables a functional assessment, although at the time of writing, no methods exist for reliably assessing protein expression and cross-reactivity from genomic data. Determining cross-reactivity is key to estimating how broadly Bexsero can cover diverse meningococcal variants, as there are only four variants that exactly match the vaccine components. These real-world data suggest that the MATS assay, a surrogate marker of the correlate of protection against N. meningitidis infection (SBA assay), predicts cross-reactivity reliably: none of the meningococci that occurred in individuals who were fully vaccinated according to schedule were predicted to be reactive. These data also support the thresholds used in the development of the MenDeVAR index (testing of ≥5 isolates by MATS and at least three-fourths being cross-reactive or not reactive). Variants with insufficient phenotyping testing are problematic, and availability of more phenotypic data will improve genotype-phenotype predictions through the MenDeVAR index. Identifying more meningococci that are known to be vaccine preventable *in vitro* and inferred *in vivo* would enable global regions to rigorously assess the breadth of vaccine coverage in their population, providing more detailed vaccine effectiveness and cost-effectiveness estimates for vaccine policy decisions.

The limitations of this study included the incomplete availability of meningococcal isolates, required for WGS analysis, although the rate observed (47.6%) was comparable to that in other studies (15). Attempts were made to optimize data from PCR-confirmed cases, but in many specimens the amount of meningococcal DNA was too small for antigen typing. Underlying immunodeficiencies among cases were not considered, as this information is not routinely collected during public health surveillance. There was a notable reduction in IMD cases across all age groups in Scotland from early 2020 continuing into 2022, compared to previous years, which was likely secondary to social distancing measures and other restrictions implemented from March 2020 in response to the coronavirus disease 2019 (COVID-19) pandemic, interrupting meningococcal transmission.

Effective and timely public health interventions are necessary for the prevention of infectious diseases, with vaccines against N. meningitidis being a highly effective intervention. The evidence obtained in the 7 years following introduction of the Bexsero vaccine for infants in Scotland provides a real-world demonstration of vaccine efficacy assessed using real-time, integrated WGS in public health. This study also demonstrates the need to characterize additional, diverse meningococci as vaccine preventable to understand the impact of the vaccine and possible modification of vaccine formulations in the future.

## MATERIALS AND METHODS

The Public Health Scotland Order 2019 in Article 9(2)(i) places an obligation on Public Health Scotland (PHS) to engage in the control of spread of infectious diseases in accordance with section 43 of the National Health Service (Scotland) Act 1978. In accordance with sections 15, 16(5), and 21(2) of the Public Health etc. (Scotland) Act 2008, PHS is obliged to process data in relation to notifiable diseases, health risk states of patients, notifiable organisms, and carrying out public health investigations, and therefore, individual patient consent is not required.

At the time of Bexsero introduction, the population of Scotland was ~5.47 million, with an annual birth cohort of ~55,000. Data for all Scottish IMD cases were captured by the Meningococcal Invasive Disease Augmented Surveillance (MIDAS) scheme, managed by Public Health Scotland and SMiRL-G. Records for children with IMD from 1 September 2015 to 30 June 2022 were retrieved with demographic information, including date of birth, number of Bexsero doses received, date of last Bexsero dose, age at IMD presentation, date of IMD diagnosis, clinical presentation, and laboratory sample identifiers. For each case, microbiological specimens were identified, mode of diagnosis was established (i.e., microbiological culture or meningococcal *ctrA* PCR), and sequence data were retrieved, where available. The PubMLST database (pubmlst.org) held isolate records, WGS data, and associated provenance information. To assess vaccination status, the number of vaccine doses received and the interval between the last vaccine dose and IMD onset was determined (Table 3). Fourteen days postvaccination was considered sufficient for vaccine-induced immunity to develop. The term “vaccine breakthrough cases” was used to describe IMD cases that occurred in children who were fully vaccinated according to schedule.

**TABLE 3:** Definitions of vaccination status^*a*^

Vaccination status	No. of doses received	Interval between Bexsero dose and date of onset of IMD	No. of children (*n* = 82)
Unvaccinated	0		48
	1	<14 days	0
Partially vaccinated according to schedule	1	14 days–2 mo	8
	1	>2 mo	3
	2	<14 days	1
	2	>9 mo	3
	3	<14 days	0
Fully vaccinated according to schedule	2	14 days–9 mo	17
	3	≥14 days	2

a Vaccination status was determined by considering the number of Bexsero doses received by age of eligibility (first dose advised at 8 weeks, second dose at 16 weeks, and booster at 12 months) and the interval between the vaccine dose and the onset of invasive meningococcal disease.

For non-culture-confirmed IMD cases, diagnosis was by molecular methods. Briefly, DNA extracts from clinical specimens (EDTA-blood or CSF) were screened for meningococcal DNA using an in-house *ctrA* quantitative reverse transcription-PCR (RT-PCR) (27). Serogroup was determined with *siaD* RT-PCR (28). Nested PCR was used for amplification of *porA* (29) and *fHbp* (30) directly from clinical samples, allowing the deduction of PorA VR1 and VR2 and the fHbp peptide. Nested PCR success was correlated with positivity of the *ctrA* RT-PCR, with extracts with high cycle threshold values (weakly positive) often failing subsequent MLST or *porA*/*fHbp* typing. NHBA and NadA typing were not routinely performed.

Where possible, typing information was deduced from WGS data, including species, strain designation, MLST and ST, CC, and BAST (14). The MenDeVAR index (16) was determined for Bexsero and Trumenba: for a given isolate, each vaccine was assigned a green, amber, red, or gray status. Green indicated that the isolate possesses an antigenic variant identical to the vaccine component. For Bexsero, this was one of fHbp1, NHBA 2, NadA 8, or PorA VR2 P1.4. For Trumenba, it was fHbp peptide 45 or 55. Amber indicated that the isolate possesses an antigenic variant found experimentally to cross-react with vaccine-derived antibodies. Red indicated that the vaccine antigen variants present in the meningococcus had all been shown to be nonreactive experimentally with vaccine-derived immune responses. Gray indicated that the meningococcus possessed antigen variants for which insufficient information was available from experimental studies to determine potential cross-reactivity. The putative cross-reactive or nonreactive antigenic variants were previously determined using MATS (21) for Bexsero-induced immune responses and MEASURE or SBA assay data for Trumenba-induced immune responses.
