# Supplementary figures and images for: Exploiting Real-Time Genomic Surveillance Data To Assess 4CMenB Meningococcal Vaccine Performance in Scotland, 2015 to 2022
Source: mBio. 2023 Apr 10;14(2):e00499-23. doi: 10.1128/mbio.00499-23 (PMC10127610; doi:10.1128/mbio.00499-23)

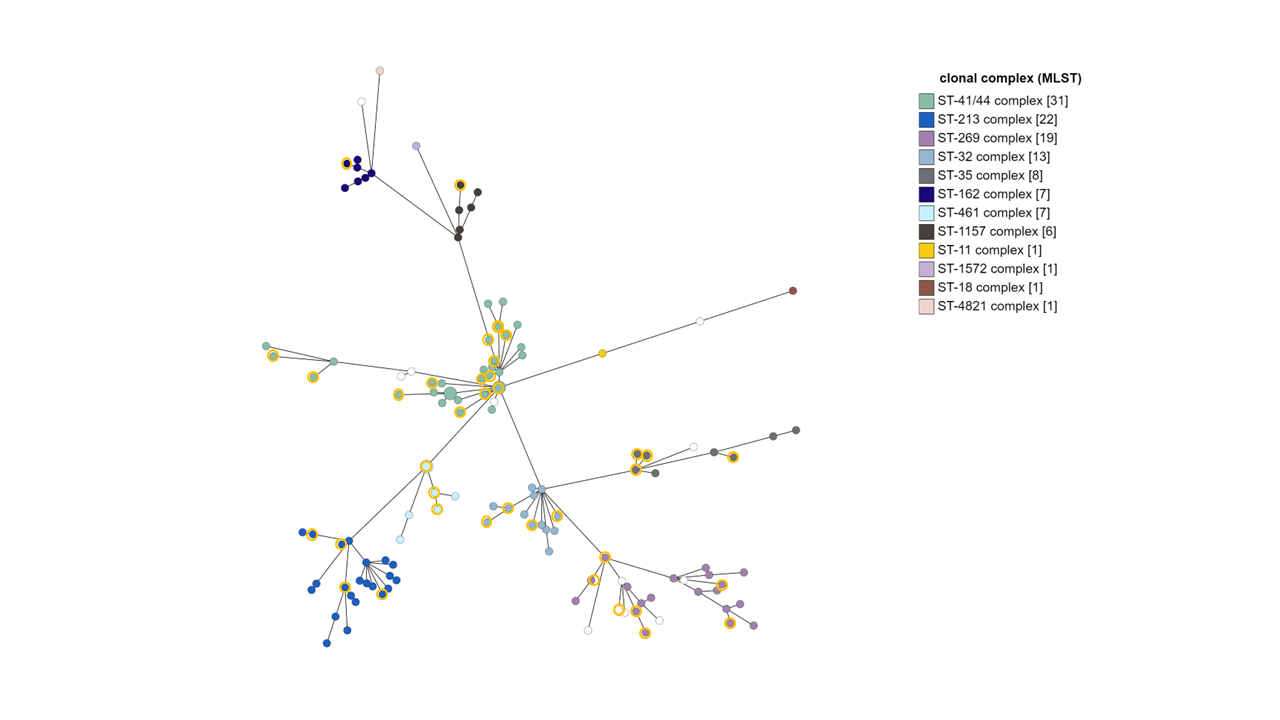

Supplement: FIG S1 [file mbio.00499-23-s0001.tif]
